# Radiologic imaging shows variable accuracy in diagnosing orbital inflammatory disease and assessing its activity

**DOI:** 10.1038/s41598-020-78830-0

**Published:** 2020-12-14

**Authors:** Min Joung Lee, Bronwyn E. Hamilton, David Pettersson, Kimberly Ogle, Jennifer Murdock, Roger A. Dailey, John D. Ng, Eric A. Steele, Rohan Verma, Stephen R. Planck, Tammy M. Martin, Dongseok Choi, James T. Rosenbaum

**Affiliations:** 1 Casey Eye Institute, Oregon Health and Science University, Portland, OR USA; 2 Department of Ophthalmology, Hallym University Sacred Heart Hospital, 22 Gwanpyeong-ro 170 beon-gil, Dongan-gu, Anyang, 14068 Republic of Korea; 3 Department of Radiology, Oregon Health and Science University, Portland, OR USA; 4 Oregon Health and Science University–Portland State University School of Public Health, Oregon Health and Science University, Portland, USA; 5 Graduate School of Dentistry, Kyung Hee University, Seoul, Republic of Korea; 6 Devers Eye Institute, Legacy Health System, Portland, OR USA; 7 Department of Medicine, Oregon Health and Science University, Portland, OR USA

**Keywords:** Diseases, Medical research

## Abstract

Radiologic orbital imaging provides important information in the diagnosis and management of orbital inflammation. However, the diagnostic value of orbital imaging is not well elucidated. This study aimed to investigate the diagnostic accuracy of orbital imaging to diagnose orbital inflammatory diseases and its ability to detect active inflammation. We collected 75 scans of 52 patients (49 computed tomography (CT) scans; 26 magnetic resonance (MR) imaging scans). Clinical diagnoses included thyroid eye disease (TED) (41 scans, 31 patients), non-specific orbital inflammation (NSOI) (22 scans, 14 patients), sarcoidosis (4 scans, 3 patients), IgG4-related ophthalmic disease (IgG4-ROD) (5 scans, 3 patients), and granulomatosis with polyangiitis (GPA) (3 scans, 1 patient). Two experienced neuroradiologists interpreted the scans, offered a most likely diagnosis, and assessed the activity of inflammation, blinded to clinical findings. The accuracy rate of radiological diagnosis compared to each clinical diagnosis was evaluated. Sensitivity and specificity in detecting active inflammation were analyzed for TED and NSOI. The accuracy rate of radiologic diagnosis was 80.0% for IgG4-ROD, 77.3% for NSOI, and 73.2% for TED. Orbital imaging could not diagnose sarcoidosis. Orbital CT had a sensitivity of 50.0% and a specificity of 75.0% to predict active TED using clinical assessment as the gold standard. The sensitivity/specificity of orbital MR was 83.3/16.7% for the detection of active NSOI. In conclusion, orbital imaging is accurate for the diagnosis of IgG4, NSOI, and TED. Further studies with a large number of cases are needed to confirm this finding, especially with regard to uncommon diseases. Orbital CT showed moderate sensitivity and good specificity for identifying active TED.

## Introduction

Orbital inflammation accounts for up to 6% of all orbital diseases and is one of the most frequent causes for orbital biopsy^1–3^. The most common cause of orbital inflammation is Graves’ disease, but various other systemic inflammatory diseases are known to be associated with orbital inflammation, such as sarcoidosis, granulomatosis with polyangiitis (GPA), IgG4-related ophthalmic disease (IgG4-ROD), and histiocytosis. Non-specific orbital inflammation (NSOI), also known as idiopathic orbital inflammation, or orbital pseudotumor, can be diagnosed after exclusion of identifiable local or systemic causes^4^.

The diagnosis of orbital inflammation can be made through a comprehensive approach considering history, clinical symptoms and signs, blood tests, and imaging studies. However, clinical symptoms and signs of orbital inflammatory diseases often overlap. Patients may present with pain, swelling, exophthalmos, reduced extraocular motility, diplopia, color vision abnormality, afferent pupillary defect, and decreased visual acuity. Various antibody tests can be used to diagnose orbital inflammation but clinicians should decide the number and type of tests to be performed based on clinical impression. Most blood tests have limited sensitivity and specificity. Exceptions include an anti-neutrophil cytoplasmic antibody test which has good specificity for GPA but limited sensitivity when GPA is confined to the orbit^5^; and thyroid stimulating immunoglobulin (TSI) which has good sensitivity and specificity for thyroid eye disease (TED)^6^. Moreover, there is no laboratory test which can be used to diagnose NSOI. Orbital biopsy is an invasive procedure which carries risk of injury to the optic nerve or extraocular muscle.

Radiologic orbital imaging plays an essential role in the diagnosis and management of orbital inflammation. However, there is a paucity of studies verifying the diagnostic value of orbital imaging. In this study, we aimed to investigate the accuracy of orbital imaging in diagnosing various types of orbital inflammation and the ability to differentiate active from inactive inflammation. We have collected orbital imaging scans of various orbital inflammatory diseases. Two neuroradiologists were asked to render a radiological diagnosis and to assess the activity of the inflammation while blinded to all clinical information. Radiological diagnosis was compared with clinical diagnosis, and radiological assessment of activity with clinical activity, as judged by clinicians.

## Results

A total of 75 imaging studies (Computerized tomography (CT): 49 scans, Magnetic resonance (MR): 26 scans) in 52 patients were reviewed. Clinical diagnoses included TED (41 scans from 31 patients), NSOI (22 scans from 14 patients), Sarcoidosis (4 scans from 3 patients), IgG4-ROD (5 scans from 3 patients), and GPA (3 scans from 1 patient). The choice of scan was made by the ordering clinician. Table 1 shows the demographics of the study population and number of imaging studies according to each group. Most of the NSOI patients (12 of 14 patients) had biopsy-confirmed diagnosis. All IgG4-ROD and GPA cases were pathologically confirmed. For sarcoidosis, 1 patient had orbital biopsy results and 2 patients had systemic sarcoidosis with orbital inflammation and simultaneous scleritis. TED was diagnosed based on biochemical evidence for thyroid dysfunction and characteristic eyelid and orbital signs. Biopsy was performed in 4 patients. One image scan was collected in 35 patients, 2 scans in 11 patients, and 3 scans in 6 patients.

**Table 1:** Demographics of patients and number of imaging studies.

Clinical diagnosis	TED	NSOI	Sarcoidosis	IgG4-ROD	GPA
Number of patients	31	14	3	3	1
Number of patients with orbital biopsy results	4	12	1	3	1
Age at first imaging, mean (SD), years	56.5 (10.6)	48.1 (17.4)	56.0 (10.2)	43.7 (15.5)	30
Gender (M:F)	12:19	2:12	2:1	2:1	1:0
Number of scans	41	22	4	5	3

*TED* thyroid eye disease, *NSOI* non-specific orbital inflammation, *IgG4-ROD* IgG4-related ophthalmic disease, *GPA* Granulomatosis with polyangiitis.

**Figure 1:**
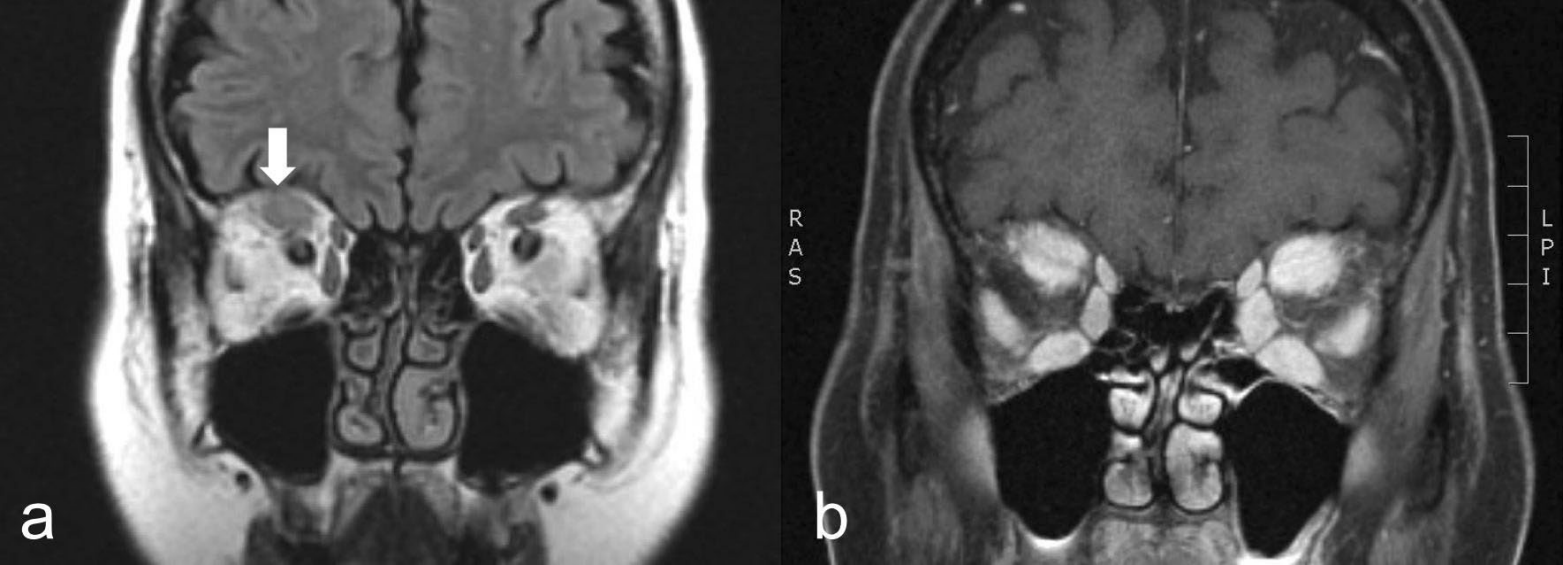
Two consecutive magnetic resonance (MR) scans of 1 patient with thyroid eye disease. (**A**) FLAIR MR image at initial visit shows enlarged superior rectus in the right orbit with slightly increased signal (white arrow). (**B**) After 8 months, fat suppressed T1-WI contrast enhanced MR of the same patient demonstrates mildly asymmetric enlargement of all extraocular muscles in both orbits with intense associated enhancement. The radiologic diagnosis for these 2 scans was non-specific orbital inflammation.

**Figure 2:**
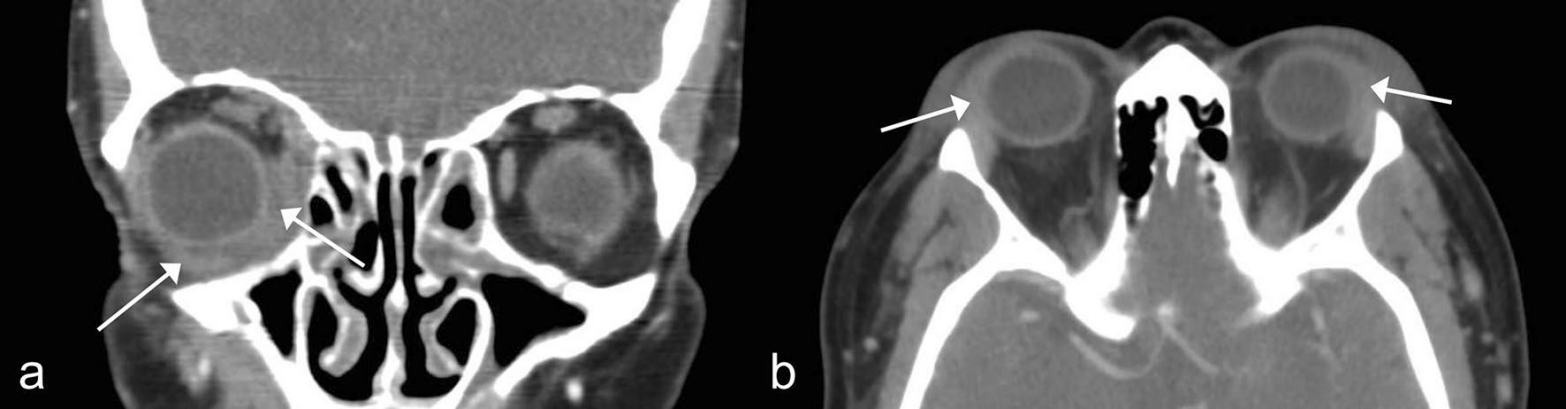
Representative images of non-specific orbital inflammation (NSOI). (**A**) Coronal, contrast-enhanced computed tomography (CT) image showed inferonasal diffuse infiltrative mass involving inferior rectus and medial rectus muscles (white arrows). The clinical and radiological diagnosis was NSOI. (**B**) Fusiform mild enlargement of both lacrimal glands shows isodensity and minimal enhancement on contrast-enhanced CT (white arrows). Preseptal soft tissue swelling is also present. The clinical diagnosis was lacrimal NSOI, compatible with the radiologic diagnosis.

**Figure 3:**
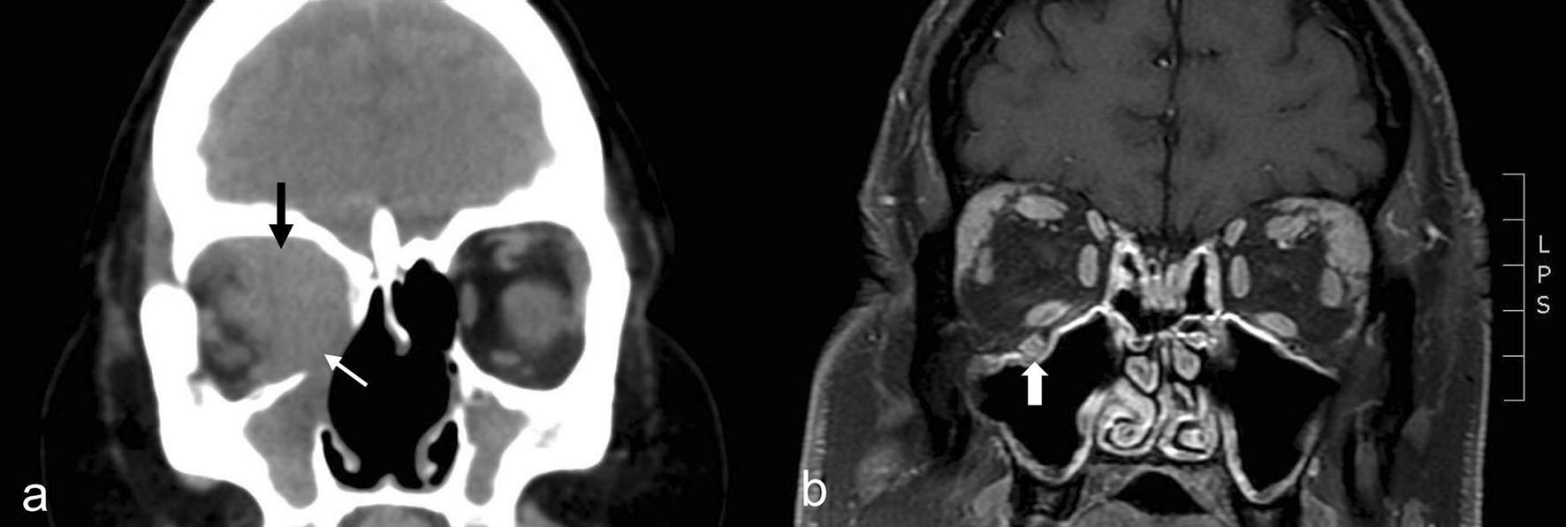
Representative images of granulomatosis with polyangiitis (GPA) and IgG4-related ophthalmic disease (IgG4-ROD). (**A**) Coronal unenhanced computed tomography scan shows diffuse, homogeneous, retrobulbar mass affecting extraconal and intraconal space of the right orbit (black arrow). Note nasal septal destruction and the contiguous sinus involvement with orbital wall and orbital floor destruction, characteristic of GPA (white arrow). The radiologic diagnosis was GPA, compatible with the clinical diagnosis. (**B**) Asymmetric, bilateral lacrimal gland enlargement is seen on coronal, fat suppressed T1-weighted contrast-enhanced magnetic resonance image. Right infraorbital nerve enlargement (white arrow) is reported as a more specific finding of IgG4-ROD, and the radiologic diagnosis was IgG4-ROD.

With a reference standard of clinical diagnosis, the accuracy rate of radiologic diagnosis was 73.2% in TED; radiologic diagnosis was correct in 30 of 41 scans as a primary diagnosis. Radiologic diagnosis was incorrect in 11 of 41 scans of TED, and the false diagnoses were NSOI in 6 scans (Fig. 1), IgG4-ROD in 4 scans, and normal in 1 scan. The accuracy rate of radiologic diagnosis for NSOI was 77.3%. Radiologic diagnosis was correct in 17 of 22 scans. In the other 5 scans, various erroneous diagnoses were made including TED, IgG4-ROD, sarcoidosis, normal, and infection (Fig. 2). Orbital imaging diagnosed GPA (100%) and IgG4-ROD (80%) with relatively high accuracy, but could not diagnose sarcoidosis (Fig. 3). The detailed radiologic diagnoses for each clinical diagnostic group are shown in Table 2.

**Table 2:** Accuracy rate of radiologic diagnosis for various orbital inflammatory diseases.

Clinical diagnosis	Accuracy rate of radiologic diagnosis (true:false)	Erroneous diagnoses details
TED n = 41	73.2 (30:11)	NSOI: 6, IgG4-ROD: 4, Normal:1
NSOI n = 22	77.3 (17:5)	TED:1, IgG4-ROD:1, Sarcoid:1, Normal:1, Infection:1
Sarcoidosis n = 4	0 (0:4)	TED:1, NSOI:1, meningioma:2
IgG4-ROD n = 5	80 (4:1)	Sarcoid:1
GPA n = 3	100 (5:0)	–

*TED* thyroid eye disease, *NSOI* non-specific orbital inflammation, *IgG4-ROD* IgG4-related ophthalmic disease, *GPA* Granulomatosis with polyangiitis.

Diagnostic performance of imaging for predicting inflammation activity was also calculated in TED and NSOI (Table 3). For predicting clinically active TED, the sensitivity of CT for detecting active TED was 50.0%, and the specificity was 75.0%. MR was performed in 7 patients with active TED, and all MR scans were correctly radiologically assessed as active disease. Regarding NSOI, 15 scans were taken in patients with active disease, while 7 studies were performed in inactive disease. CT showed 66.7% sensitivity and 0% specificity while MRI showed 83.3% sensitivity and 16.7% specificity for the detection of active inflammation in NSOI.

**Table 3:** Sensitivity and specificity of the imaging study for assessment of inflammation activity in TED and NSOI.

		TED (n = 41)		Sensitivity (95% CI)	Specificity (95% CI)
		Clinically active	Clinically inactive		
CT (n = 34)	Active	9	4	50.0	75.0
	Inactive	9	12	(26.7–73.2)	(47.4–91.7)
MR (n = 7)	Active	7	0	100	–
	Inactive	0	0	(56.1–100)	

		NSOI (n = 22)		Sensitivity (95% CI)	Specificity (95% CI)
		Clinically active	Clinically inactive		
CT (n = 10)	Active	6	1	66.7	0
	Inactive	3	0	(29.9–92.5)	(0–97.5)
MR (n = 12)	Active	5	5	83.3	16.7
	Inactive	1	1	(35.9–99.6)	(8.8–63.5)

*TED* thyroid eye disease, *NSOI* non-specific orbital inflammation.

## Discussion

Orbital inflammation can have a wide range of underlying etiologies. With the recent evolution of molecular targeted therapies such as teprotumumab or tocilizumab for TED^7,8^, and rituximab for GPA^9^, identifying the underlying cause of orbital inflammation is becoming more important. Radiologic imaging is commonly used in an ophthalmology clinic to verify the causes of orbital inflammation, and many clinicians make a diagnosis deferring to the interpretation of a radiologist. However, it is unknown how accurately a radiologist can assess the cause of orbital swelling on orbital imaging study. We believe that this is the first report to analyze quantitatively the value of radiologic imaging for the diagnosis of orbital inflammation. Our study indicates that the accuracy rate varies depending on the disease entity. Orbital imaging precisely diagnosed GPA and IgG4-ROD with characteristic findings^10^, although the number of scans was small. Radiologic orbital imaging is highly accurate for the diagnosis of TED and NSOI.

In this study, the radiologists were able to diagnose TED with an accuracy of 73.2%. There are some previously well-known radiologic characteristics of TED including bilateral tendon-sparing extra-ocular muscle (EOM) enlargement, orbital fat expansion, proptosis, and/or lacrimal gland (LG) enlargement^11–14^. Radiologic imaging is not usually essential for the diagnosis of TED because TED has discriminating ophthalmic characteristics such as eyelid retraction and lid lag. However, it is sometimes difficult to be diagnosed if the eyelid signs are not evident or thyroid function tests are normal. A high accuracy rate of orbital imaging suggests that an imaging study can be a valuable tool for the diagnosis of atypical TED. In addition to establishing a diagnosis, imaging studies play a valuable role in tracking disease progression. Among 31 patients, TSI was tested in 14 patients; TSI was detected in 12 patients, and not detected in 2 patients who had chronic inactive TED. Radiologic diagnosis was correct even in these 2 patients. The radiologic diagnosis was incorrect in 11 scans and the most common erroneous diagnoses were NSOI and IgG4-ROD. These diagnoses were made especially when EOM tendon sparing was not significant or EOM involvement pattern was atypical. Although tendon-sparing EOM enlargement is one of the typical findings for TED, it is not a pathognomonic or diagnostic feature. Simon et al.^15^ evaluated the configuration or EOM in patients of TED and reported 6.4% of patients showed tendon involvement on CT or MR.

NSOI is one of the most elusive orbital diseases. Identifying NSOI radiologically is especially challenging because of the heterogeneity of the disease as shown by our prior studies on gene expression in orbital tissue^16,17^. The term NSOI is applied when a patient having orbital inflammation has a disease that defies categorization despite biopsy. Making a diagnosis of NSOI is frequently challenging because of the heterogenous clinical presentations, obscure etiology, and lack of specific biomarkers. Generally, an infiltrative mass and poorly defined mass with moderate diffuse enhancement are characteristic findings on contrast-enhanced CT. Hypo-intensity on both T1-weighted image (WI)/T2-WI on MR are typical because of the cellular infiltration and fibrosis^12,18^. Hyperintensity on T2-WI is expected for active inflammatory diseases, however some inflammatory disease could be misleading due to the presence of fibrosing components that will present as hypo-intensity on T2-WI. However, there are no definite diagnostic imaging findings for NSOI that can serve to distinguish it from other orbital inflammatory diseases. In this study, the radiologic diagnosis was correct in 17 of 22 NSOI scans and the erroneous diagnoses for the other 5 scans were variable. Lacrimal NSOI was misdiagnosed as sarcoidosis in bilateral cases, and as TED or normal in mild cases. One diffuse NSOI was mistaken for IgG4-ROD when the inflammation extensively affected the orbit.

IgG4-ROD was fairly accurately diagnosed by imaging study. The radiologists made a diagnosis of IgG4-ROD in 4 patients, and sarcoid in 1 patient. Diffuse enlargement of LG, EOM enlargement, orbital fat infiltration, and trigeminal nerve enlargement are common radiologic findings of IgG4-ROD. The findings of LG enlargement and trigeminal nerve enlargement, however, could be seen with other diseases^19–24^. Bilaterality has been reported as 62–76%^19–21^. Extra-ophthalmic involvement such as parotid gland enlargement, cervical lymph node enlargement, and paranasal sinus mucosal thickening also can be detected in orbital imaging. In our case series, all 5 scans of IgG4-ROD presented with bilateral LG enlargement. Three of them showed simultaneous parotid gland enlargement, which led the radiologists to favor the diagnoses of sarcoidosis in one case. Sarcoidosis might be aided by review of contemporaneous imaging studies, including chest CT, which has more specific features. Although LG is the most frequently involved anatomical site in IgG4-ROD, there are many orbital conditions that can show bilateral LG presentations. Tang et al.^25^ reported diagnoses of 95 patients with bilateral LG disease. The most prevalent diagnosis was NSOI followed by sarcoidosis, LG prolapse, lymphoma, lymphoid hyperplasia, and dacryops. Infraorbital nerve enlargement was suggested as a specific sign of IgG4-ROD by several researchers^26–28^. There was only 1 scan that showed mild, unilateral infraorbital nerve enlargement in our image set.

Assessment of TED activity is clinically important in terms of establishing a treatment strategy. Patients in the active phase are usually considered as candidates for immunomodulation, whereas rehabilitation surgeries are more suitable for patients in the inactive phase. CAS (clinical activity score) is a well-established clinical scoring system to evaluate TED activity, but it is subjective and has limited power to discriminate those who do and do not respond to immunomodulating treatment^29^. Our study showed orbital CT had moderate sensitivity and high specificity for detecting active inflammation in TED. This may reflect the fact that there are no established CT characteristics for active TED. Some researchers reported the volume or density of orbital fat, EOM, and LG were different according to the activity of TED^13,30^. We tried to compare sensitivity and specificity between CT and MR. However, we could not validate the ability of MR to discriminate between active and inactive TED because all MRs were performed in patients with active TED.

This report has several limitations. This is retrospective study from a single center, and our numbers were limited, especially in groups of relatively rare diseases such as IgG4-ROD, sarcoidosis, and GPA. Our sample size is too small to compare the diagnostic value of MR versus CT for evaluating orbital inflammation activity. Orbital infection and diffuse infiltrative malignancies such as lymphoma and metastatic tumors also should be included in this case series because they are important diagnoses that need to be differentiated from orbital inflammation in the clinic. We are in the process of collecting more images with an extended spectrum of diagnoses. Our results rely on two experienced radiologists’ capability. Finally, calculating sensitivity and specificity requires a gold standard. Clinical impression was used here in conjunction with histopathological confirmation in some but not all cases. Furthermore, no such standard has been validated for disease activity. Currently we can only compare the radiologists’ impression to that of the clinician.

Our study includes several novel findings. Radiologic orbital imaging is fairly accurate for the diagnosis of several orbital inflammatory diseases, including IgG4-ROD (80%), NSOI (77.3%) and TED (73.2%). However, the radiologists failed to derive the correct diagnosis in 20–30% of image scans. These results indicated that even an experienced neuroradiologist may struggle to make a precise diagnosis in some subsets of orbital inflammation based on only imaging without any clinical information. Reasons for this likely include a lower frequency of the non-TED diagnoses. This is also certainly far from actual clinical practice; a radiologist usually renders a diagnosis integrating clinical information. However, radiologic impression plays a key role to make a diagnosis when clinical data are ambiguous and laboratory tests are inconclusive. Our results provide valuable information about the diagnostic accuracy of radiologic imaging for several orbital inflammatory diseases. Orbital CT showed moderate sensitivity and good specificity for predicting active TED. For NSOI, orbital MR showed high sensitivity and low specificity for detecting active inflammation. These findings can be reinforced by future studies collecting more scans with a wider spectrum of diagnoses and serially over time.

## Patients and methods

This study adhered to the tenets of the Declaration of Helsinki and the protocol was approved by the Institutional Review Board at Oregon Health and Science University (IRB00006301). The requirement for written informed consent was waived by Oregon Health and Science University Institutional Review Board due to the retrospective nature of the research. We collected CT and MR orbital scans, demographic and clinical data of patients who were diagnosed with various orbital inflammatory diseases from a single tertiary institute (OHSU). Orbit MR was performed by using a head coil. The MR protocol included coronal and axial T1-WI and T2-WI sequences with and without fat suppression. Diffusion-weighted images were obtained. T1-WI sequences with fat suppression were performed after contrast medium administration.

Two experienced neuroradiologists (BEH and DP) reviewed orbital imaging blinded to all demographic, clinical, or laboratory data. They were only told that the set of imaging exams included all causes of orbital diseases. The neuroradiologists interpreted images, offered a diagnosis solely based on the radiologic findings, and this diagnosis was defined as a radiologic diagnosis. The radiologists were requested to make one most likely diagnosis and the final diagnosis was made by consensus. The inflammatory activity was also interpreted and marked as ‘active’ or ‘inactive’. The radiologists had no formal training session with sample cases to reflect actual practice, however they formalized criteria for common diagnoses below for consistency prior to image review.

The radiologists analyzed the orbital images systematically and made a diagnosis based on EOM enlargement, LG enlargement, orbital fat excess, orbital apex crowding, proptosis, trigeminal nerve involvement, and adjacent sinus involvement. The detailed items evaluated were listed in Table 4. Maximum diameters of 4 rectus muscles and superior oblique muscle were measured to assess EOM enlargement. These were required to exceed 2 standard deviations above normal to be considered enlarged, based on published normative data, and typical patterns of involvement were used to establish diagnosis^31^. Tendon sparing was also checked in cases with muscle enlargement and considered indicative of TED if other imaging findings aligned and tendon thickening as indicative of NSOI if other imaging findings correlated. Orbital apex crowding was evaluated by a grade scale of fat effacement and optic nerve sheath diameter measured at its retrobulbar and waist portions, also contributed to TED diagnosis^31^.

**Table 4:** Measurement items for the analysis of orbital diseases on imaging.

	Measurements
Extraocular muscles	Inferior rectus muscle enlargement (> 6.5 mm)
	Medial rectus muscle enlargement (> 5.1 mm)
	Lateral rectus muscle enlargement (> 4.1 mm)
	Superior rectus muscle complex enlargement (> 5.2 mm)
	Superior oblique muscle enlargement (> 3.2 mm)
	Fatty infiltration of extraocular muscles
Lacrimal gland	Lacrimal gland enlargement
Orbital fat	Excess orbital fat
	Orbital fat stranding
Orbital apex and optic nerve	Orbital apex fat effacement grading 0 none; 1 (1–25%); 2 (25–50%); 3 (> 50%)
	Retrobulbar optic nerve sheath complex (> 7.1 mm)
	Waist portion of optic nerve sheath complex (> 5.4 mm)
Proptosis	CT-based exophthalmometry using lateral to medial orbital rims-corneal surface in the axial plane
Superior ophthalmic vein	Superior ophthalmic vein enlargement (> 2 mm axial or > 3 mm coronal)
Trigeminal nerve	Infraorbital nerve enlargement (> 2.0 mm)
	Any trigeminal nerve involvement; comment
Sinus	Ipsilateral/adjacent involved sinus disease 0 = none; 1 = trace; 2 = trace to 25%, 3 = 25–50%; 4 = > 50%
Signal characteristics	Enhancement pattern
	Signal intensity on T2-weighted images on magnetic resonance images

Proptosis was measured by the height of the perpendicular line from the line between the lateral orbital rim and the medial orbital rim to the posterior surface of the cornea on the axial plane that bisects the lens^32^. Symmetric proptosis and subjective fat accumulation were used to suggest a diagnosis of TED in patients who did not have typical EOM enlargement and also lacked other specific findings to suggest an alternative diagnosis. The inflammation activity was evaluated by the enhancement pattern, inflammatory fat stranding, and signal intensity on T2-weighted images on MR. T2 hyperintensity on MR images was scored on a 3 point scale of increasing intensity, and considered the most reliable finding for active inflammation by radiologists.

Findings used to establish a diagnosis of IgG4-related disease included assessment of orbital findings of inflammation involving any compartment, including EOM enlargement, with the latter involvement being asymmetrical or atypical in patten for TED, plus identifying extraorbital manifestations suggestive of IgG4-related disease. These included enlargement and enhancement of any division of the trigeminal nerves, intracranial pachymeningeal thickening and enhancement, and/or adjacent paranasal sinus inflammatory mucosal thickening, graded by degree of involvement^27,31^.

The diagnosis of GPA was established by typical findings of paranasal sinus inflammatory soft tissue thickening in association with marked thickening and/or sclerosis of paranasal sinus wall and/or overt osseous destruction. Orbital soft tissue findings were expected to be contiguous with the areas of sinus involvement in GPA^17,33^. Imaging criteria suggested for sarcoidosis included lacrimal gland enlargement and enhancement in conjunction with parotid and/or cervical adenopathy that did not fit one of the other patterns of inflammatory diseases, given that no specific criteria are not established for this rare diagnosis.

Clinical diagnosis was the reference standard and was made according to the diagnostic criteria relevant to each diagnosis. (1) TED: biochemical thyroid dysfunction and the presence of any characteristic clinical sign such as eyelid retraction, exophthalmos, EOM involvement, and optic nerve dysfunction. (2) NSOI: clinical signs and symptoms suggestive of orbital inflammation without evidence of systemic disease association, and/or histopathological patterns of NSOI such as nonspecific polymorphous infiltrate of well-differentiated lymphocytes, plasma cells, neutrophils, and eosinophilic granulocytes with various degree of fibrosis, when confirmed by orbital biopsy. (3) Sarcoidosis: clinical signs of orbital inflammation; clinico-radiological evidence of systemic sarcoidosis, supported by histologic evidence from any site in the body of noncaseating epithelioid-cell granuloma in the absence of organisms or foreign body^34^. (4) GPA: clinical signs of orbital inflammation, anti-neutrophil cytoplasmic antibody positive, and pathologic evidence of granulomatous necrotizing vasculitis in orbit or another organ. (5) IgG4-ROD: clinical examination suggesting diffuse/localized swelling or masses in the orbital region, elevated serum IgG4, and/or histopathological findings of marked lymphocyte/plasmacyte infiltration and fibrosis with infiltration of IgG4 + plasma cells^35^.

Data about clinical activity of inflammation were collected in patients with TED or NSOI by referring to the medical records at the time of imaging. TED activity was assessed according to the CAS, and cases with CAS ≥ 3 were considered as having active TED^36^. For NSOI, the activity of inflammation was assessed based on the physician’s judgment. NSOI with profound pain and obvious soft tissue inflammation signs prompting immunosuppressive treatment was considered as active NSOI. NSOI was considered as inactive when the patient had no definite clinical evidence of inflammation after systemic immunosuppression had been stopped or when minimal dose of immunosuppressive agent was maintained in order to prevent recurrence without any sign of inflammation.

The accuracy rate of radiologic diagnosis was calculated for each diagnostic entity. The accuracy rate was defined as the proportion of the scans for which the radiologist made the right diagnosis divided by the sum of correct and incorrect diagnoses diagnosis. The assessment of inflammatory activity was summarized in a two-by-two frequency table and thereby sensitivity and specificity were derived. All calculations were carried out using SPSS (ver 25.0; IBM SPSS Statistics, Armonk, NY, USA).

## Data Availability

The datasets used and/or analyzed in this study are available from the corresponding author on reasonalble request.
